# An Unusual Case of Group B Streptococcal Meningitis with Concomitant Varicella-Zoster Virus Infection in a Previously Healthy Male

**DOI:** 10.7759/cureus.32134

**Published:** 2022-12-02

**Authors:** Farshad Shahkarami, Mehrnaz Fallah Tafti, Mahbobeh Alizadeh, Alireza Foroughi, Roozbeh Bayati

**Affiliations:** 1 Medical Student, Tehran University of Medical Sciences, Tehran, IRN; 2 Neurology, Tehran University of Medical Sciences, Tehran, IRN; 3 Infectious Diseases, Nosocomial Infections, Tehran University of Medical Sciences, Tehran, IRN; 4 Radiology, Tehran University of Medical Sciences, Tehran, IRN; 5 Infectious Diseases, Tehran University of Medical Sciences, Tehran, IRN

**Keywords:** meningoencephalitis, meningitis, shingles, varicella-zoster virus, streptococcus agalactiae, group b streptococcus

## Abstract

Group B *Streptococcus *(GBS) is a well-known organism that can be part of the normal gastrointestinal and genital tract flora. However, it can cause various infections, mostly in neonates, pregnant women, and patients with predisposing factors. Meningitis caused by GBS, though common in neonates, is uncommon in adults, especially those with no previous health issues. Here, we present an unusual case of GBS meningitis in a 22-year-old previously healthy man. He came to the emergency room with complaints of acute headache and an altered level of consciousness, and his cerebrospinal fluid analysis was consistent with bacterial meningitis due to *Streptococcus agalactiae*. Later, vesicular lesions on his face caused by varicella-zoster virus (VZV) infection complicated his situation. He received intravenous ceftriaxone and dexamethasone and oral acyclovir. He had a complete recovery and was discharged without any sequelae. Though uncommon, this serious condition needs prompt diagnosis and treatment due to its high mortality rate. To our knowledge, this is one of the few known cases of GBS meningitis in a previously healthy adult and the only one to report VZV infection as a possible complication.

## Introduction

Streptococci have been divided into several groups based on Lancefield and hemolytic groupings. Group B *Streptococcus *(GBS), also known as *Streptococcus agalactiae*, is a well-known, gram-positive, catalase-negative pathogen with beta-hemolytic features. It can be part of the normal flora of the gastrointestinal and genital tract in both males and females.

Although GBS infections usually present acutely in neonates and pregnant women, they can sometimes affect non-pregnant adults, especially the elderly and those who have underlying conditions such as obesity.

Recent studies have shown that GBS infections among non-pregnant adults have increased, and their incidence was estimated to be approximately 11 cases per 100,000 population in 2016 [1].

GBS infections usually present as skin and soft-tissue infections, bacteremia without a clear source, lower respiratory tract infections, urinary tract infections, etc. [2]. Meningitis remains one of the less common presentations of GBS infection, and its prevalence among invasive cases has been estimated to be 4% in population-based studies in 1991 and 1993 and 1.1% in 2016 [1-3].

Unfortunately, it has a relatively high mortality rate. In a literature review that included 141 patients with community-acquired GBS meningitis, 31% of the patients died, most of whom were immunocompromised or had advanced age [4].

Patients with predisposing factors (diabetes mellitus, immunodeficiency, malignancies, cardiovascular diseases, chronic obstructive pulmonary disease, liver or renal diseases, etc.) are at a higher risk for GBS infections. In adult cases, most patients (88.0%) have at least one underlying medical condition [5]. However, less commonly, non-pregnant adults with no risk factors can be affected by GBS infections, in addition, bacterial meningitis due to GBS is rare in previously healthy patients.

On the other hand, infection with herpes zoster is common. According to the Centers for Disease Control and Prevention, approximately one-third of the population experiences herpes zoster during their lifetime. The incidence of herpes zoster infection has increased from 2.5/1,000 in 1993 to 7.2/1,000 in 2016 (among adults aged ≥35 years) [6].

Herpes zoster often results from the reactivation of latent varicella-zoster virus (VZV) that is usually dormant in the dorsal root of sensory ganglia by a particular cell-mediated immunity [7].

There are several risk factors for VZV infection or reactivation, including female gender, old age, physical trauma, and comorbid conditions such as immunosuppression, malignancies, autoimmune diseases, or chronic kidney and lung disease [8].

Herpes zoster infections usually present as rash and acute neuritis, but complications can affect many organs, including the central nervous system. Aseptic meningitis has been reported in a subset of immunocompetent patients affected by VZV [9]. However, the concurrent incidence of bacterial meningitis and VZV infection is an area less explored.

## Case presentation

A 22-year-old man came to the emergency department with an acute headache and an altered level of consciousness. His symptoms had started one night ago after smoking a water pipe. He had taken acetaminophen tablets at home. Because the symptoms worsened by the morning, he came to the hospital. He did not mention using any other medications recently. He did not have comorbidities such as an immunocompromised status or chronic diseases.

He did not report any recent head trauma, but he had a history of head and eye injuries due to a car accident five years ago, which resulted in unilateral blindness. His medical history was otherwise unremarkable. He had not received any coronavirus disease 2019 and herpes zoster vaccines.

On arrival at the emergency department, he had the following vital signs: forehead (temporal) temperature (by digital thermometer), 38.9°C; blood pressure, 100/68 mmHg; pulse rate, 123 beats/minute; respiratory rate, 19 breaths/minute; and oxygen saturation, 96% at rest in room air.

He was agitated, and the Glasgow Coma Scale (GCS) score was 13 out of 15. His left eye was prosthetic. He also had nuchal rigidity. There were no other positive findings on his physical examinations; there were no signs of infection on his face, he did not have Kernig or Brudzinski signs, and the deep tendon reflexes were normal.

Approximately an hour after admission, he had an episode of generalized tonic-clonic seizure that was immediately controlled with 10 mg of intravenous (IV) diazepam. Subsequently, his temporal temperature increased to 40.7°C, and he became lethargic.

Because of clinical suspicion of intoxications, toxicology screening tests were sent to the laboratory. The urinary screen was negative for common causes of intoxication and drug poisoning that could cause seizures a few hours after drug administration (tricyclic antidepressants, anticonvulsants, etc.).

To rule out meningoencephalitis, he underwent a lumbar puncture. The cerebrospinal fluid (CSF) was turbid, and the analysis showed a white blood cell (WBC) count of 5,500 (lymphocytes: 10%, neutrophils: 90%), a glucose level of 10 mg/dL, and a protein level of 259 mg/dL. Because all findings were in favor of bacterial meningitis, empirical IV antibiotic therapy was initiated with 2 g of ceftriaxone and 1 g of vancomycin twice a day. In addition, 8 mg of dexamethasone was administered before the antibiotic injection and was continued for four days. Three days after admission, the CSF culture was positive for *Streptococcus agalactiae*, and based on the antibiogram results, vancomycin was discontinued, and ceftriaxone was continued for two weeks.

**Figure 1:**
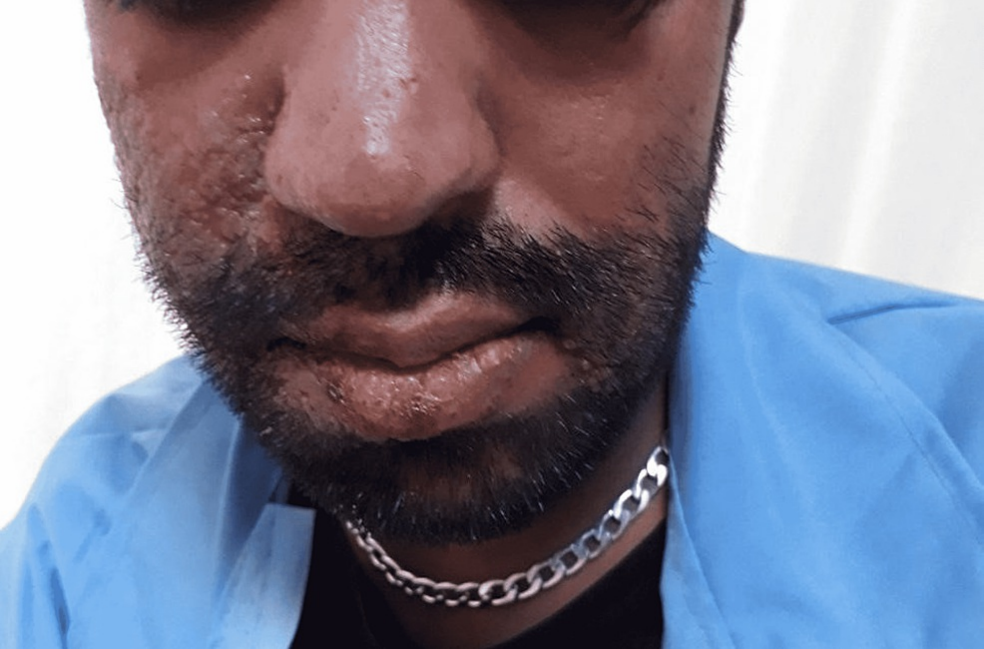
Fourth day of admission.

**Figure 2:**
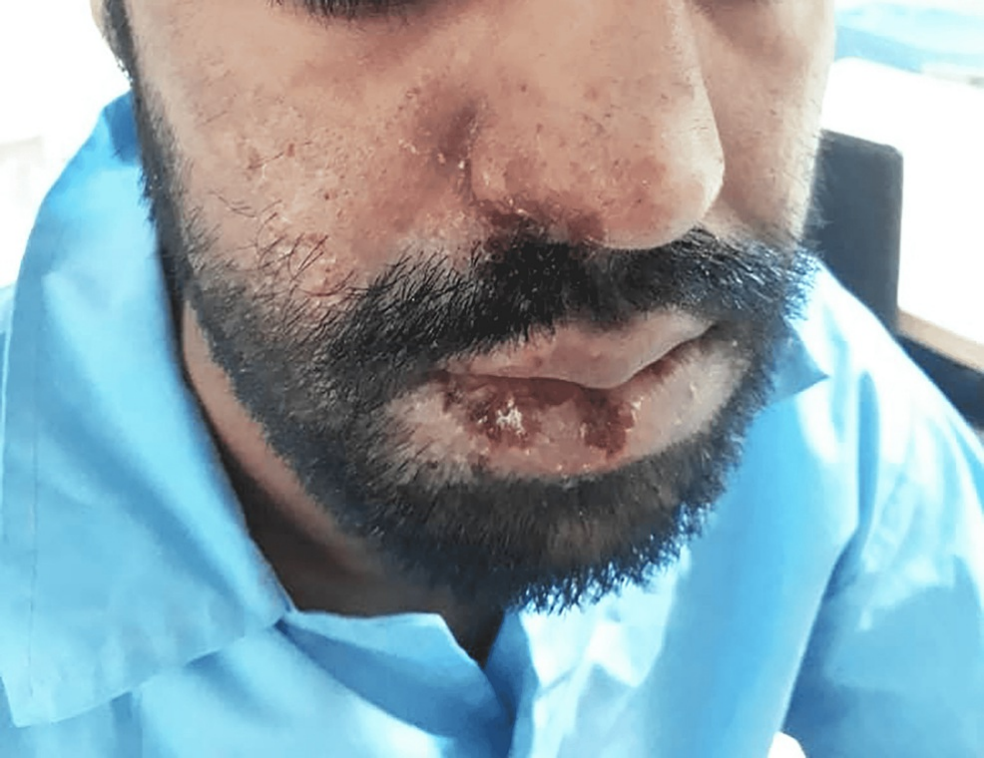
Eighth day of admission.

On the following day, his level of consciousness deteriorated, and the GCS score declined to 11 out of 15. On the third day of admission, he developed vesicular lesions on the right side of his face (lips, nasolabial, and buccal regions), which were diagnosed as shingles (Figures 1, 2). He was treated with oral acyclovir (800 mg every five hours for seven days).

To rule out other possible sites of infection and investigate likely predisposing factors for his condition, more paraclinical tests were performed.

His abdominopelvic sonography and spiral chest computed tomography (CT) scan did not reveal any unusual findings. The electrocardiogram was normal, and the echocardiogram revealed no sign of endocarditis. Additionally, serum immunoglobulins and complement proteins were normal, and the enzyme-linked immunosorbent assay test was negative for anti-HIV 1, 2.

**Figure 3:**
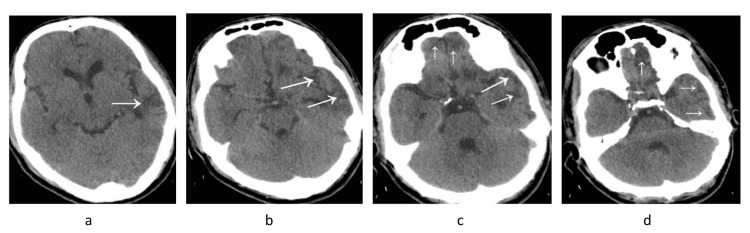
Brain computed tomography scan. Cortical hypodensity in the left temporal lobe (a-d) next to the temporal bone and around the Sylvian fissure of the same side (b) as well as at the inferior aspect of the frontal lobes bilaterally (c, d).

On his brain CT scan, there were no signs of CSF leakage. However, it revealed cortical hypodensity in the left temporal lobe next to the temporal bone and around the Sylvian fissure on the same side. There were also signs of bilaterally cortical hypodensity at the inferior aspect of the frontal lobes (Figure 3). All these findings were in favor of meningitis and adjacent cortical encephalitis. Because the patient did not have insurance coverage and was improving clinically, brain magnetic resonance imaging and electroencephalography were not performed to avoid imposing additional charges. The patient’s laboratory data are displayed in Table 1.

**Table 1:** Laboratory data *Simultaneous blood sugar: 138 mg/dL. WBC: white blood cell; Hb: hemoglobin; Plt: platelet; CRP: C-reactive protein; BUN: blood urea nitrogen; Cr: creatinine; PTT: partial thromboplastin time; PT: prothrombin time; INR: international normalized ratio; AST: aspartate aminotransferase; ALT: alanine aminotransferase; ALP: alkaline phosphatase; CSF: cerebrospinal fluid; RBC: red blood cell; LDH: lactate dehydrogenase; TCA: tricyclic antidepressant; VBG: venous blood gases; CPK: creatine phosphokinase

Lab/Date	WBC (mm^3^)	Hb (g/dL)	Plt (mm^3^)	CRP (mg/L)	BUN (mg/dL)	Cr (mg/dL)	Na (meq/L)	K (meq/L)	BS (mg/dL)
Day 1	39.9	16.6	310	20.4	24	1.03	141	3.7	123
Day 3	28.8	13.1	223	-	36	0.76	145	4.0	124
Day 4	19.5	13.5	184	73.2	32	0.90	141	4.0	121
Day 5	9.2	14.7	219	36.9	32	1	141	4.8	-
Day 14	9.0	14.1	257	7.3	32	1.1	143	4.3	-
Lab/Date	Ca (mg/dL)	Ph (mg/dL)	PTT (seconds)	PT (seconds)	INR	AST (IU/L)	ALT (IU/L)	ALP (IU/L)	Mg (mg/dL)
Day 1	10.5	1.5	30	13	1.2	33	27	164	2.5
CSF analysis and culture	Appearance	WBC	Neutrophil	Lymphocyte	RBC	Glucose (mg/dL)	Protein (mg/dL)	LDH (IU/L)	
	Turbid	5,500	90%	10%	450	10*	259	110	
	Culture	Streptococcus agalactiae		Sensitive: penicillin, ampicillin, ceftriaxone, cefepime, vancomycin					
				Intermediate: clindamycin					
				Resistance: not reported					
Toxicology	Negative for amphetamines, methamphetamines, cocaine, morphine, methadone, barbiturates, TCAs, buprenorphine, tramadol, benzodiazepines								

Four days after admission, his level of consciousness improved, and his GCS score was 15 out of 15. After two weeks of hospitalization, the patient was discharged without any signs or symptoms. After two months, an infectious disease specialist and a neurologist examined him during his follow-up visit. He did not have any neurological sequels, such as seizures, impaired mental status, and hearing loss, which are common complications of bacterial meningitis.

## Discussion

In this case, the patient was diagnosed with bacterial meningitis caused by *Streptococcus agalactiae* and was treated with appropriate antibiotics and corticosteroids without any complications. This case has two unusual aspects.

First, bacterial meningitis was caused by *Streptococcus agalactiae* in a patient with no predisposing factors and no verified source of infection. This is an uncommon condition. Although GBS meningitis usually occurs in neonates, sometimes it occurs in adults, usually in pregnant women or patients with underlying diseases. The incidence of GBS meningitis in adults has increased in the past two decades [1]. There have been reports of GBS meningitis in adult patients. Many of these cases were associated with being immunocompromised or having a confirmed extra-meningeal source of infection, but GBS meningitis in previously healthy patients with no confirmed foci of infection is less common. However, there have been reports of invasive GBS infections, including meningitis, in previously healthy adults [10-14]. Invasive GBS infections in this group, although uncommon, need immediate diagnosis and treatment due to their high mortality and morbidity rates [4].

Second, the patient had concomitant VZV infection. There have been case reports of bacterial meningitis caused by GBS, but, to our knowledge, there are no previous reports of concomitant shingles in these patients. The patient’s lesions developed two days after admission, which can be an indicator of fulminant disease that caused the patient a great amount of stress. He did not have any risk factors for VZV infection. Treatment with corticosteroids can increase the risk of VZV infection or reactivation [15,16]. However, because he had only received two days of dexamethasone before the appearance of skin lesions, it is unlikely that it made him susceptible to the virus. It has also been hypothesized that a history of head trauma in the previous month can be a risk factor for VZV infection [17]. However, because our patient’s trauma happened five years ago, it probably did not contribute to his condition. It is well known that severe medical conditions and psychological stress can be risk factors for herpes zoster infections [7]. In patients affected by bacterial meningitis, herpes reactivation can occur as well. In a study of 301 patients with bacterial meningitis, 40 (13.3%) patients developed herpes reactivation, 10 (3.3%) of whom had clinical manifestations of VZV infections, while the rest (10%) had manifestations of HSV infections [18]. However, herpes reactivation in patients with bacterial meningitis due to *Streptococcus agalactiae* is an area less studied.

## Conclusions

Invasive infections with *Streptococcus agalactiae* are becoming an increasingly recognized condition, which affects immunocompromised patients more often. Severe manifestations of GBS infections, such as meningitis, are uncommon in previously healthy patients, and concomitant with VZV reactivation is a very rare condition. This condition is important and needs immediate diagnosis and aggressive treatment because it is associated with a high rate of mortality and morbidity.
